# Deuterium MR spectroscopy: potential applications in oncology research

**DOI:** 10.1093/bjro/tzae019

**Published:** 2024-08-05

**Authors:** Almir Galvão Vieira Bitencourt, Arka Bhowmik, Eduardo Flavio De Lacerda Marcal Filho, Roberto Lo Gullo, Yousef Mazaheri, Panagiotis Kapetas, Sarah Eskreis-Winkler, Robert Young, Katja Pinker, Sunitha B Thakur

**Affiliations:** Imaging Department, A. C. Camargo Cancer Center, São Paulo, 01525-001, Brazil; Diagnósticos da América S.A., São Paulo, 04321-120, Brazil; Department of Radiology, Memorial Sloan Kettering Cancer Center, New York, NY, 10065, United States; Imaging Department, A. C. Camargo Cancer Center, São Paulo, 01525-001, Brazil; Diagnósticos da América S.A., São Paulo, 04321-120, Brazil; Department of Radiology, Memorial Sloan Kettering Cancer Center, New York, NY, 10065, United States; Department of Radiology, Memorial Sloan Kettering Cancer Center, New York, NY, 10065, United States; Department of Medical Physics, Memorial Sloan Kettering Cancer Center, New York, NY, 10065, United States; Department of Radiology, Memorial Sloan Kettering Cancer Center, New York, NY, 10065, United States; Department of Biomedical Imaging and Image-Guided Therapy, Medical University of Vienna, 1090 Vienna, Austria; Department of Radiology, Memorial Sloan Kettering Cancer Center, New York, NY, 10065, United States; Department of Radiology, Memorial Sloan Kettering Cancer Center, New York, NY, 10065, United States; Department of Radiology, Memorial Sloan Kettering Cancer Center, New York, NY, 10065, United States; Department of Radiology, Memorial Sloan Kettering Cancer Center, New York, NY, 10065, United States; Department of Medical Physics, Memorial Sloan Kettering Cancer Center, New York, NY, 10065, United States

**Keywords:** deuterium MR spectroscopy, deuterium MRI, deuterium metabolic imaging, cancer metabolism, cancer diagnosis, treatment response, IDH mutations

## Abstract

Metabolic imaging in clinical practice has long relied on PET with fluorodeoxyglucose (FDG), a radioactive tracer. However, this conventional method presents inherent limitations such as exposure to ionizing radiation and potential diagnostic uncertainties, particularly in organs with heightened glucose uptake like the brain. This review underscores the transformative potential of traditional deuterium MR spectroscopy (MRS) when integrated with gradient techniques, culminating in an advanced metabolic imaging modality known as deuterium MRI (DMRI). While recent advancements in hyperpolarized MRS hold promise for metabolic analysis, their widespread clinical usage is hindered by cost constraints and the availability of hyperpolarizer devices or facilities. DMRI, also denoted as deuterium metabolic imaging (DMI), represents a pioneering, single-shot, and noninvasive paradigm that fuses conventional MRS with nonradioactive deuterium-labelled substrates. Extensively tested in animal models and patient cohorts, particularly in cases of brain tumours, DMI's standout feature lies in its seamless integration into standard clinical MRI scanners, necessitating only minor adjustments such as radiofrequency coil tuning to the deuterium frequency. DMRI emerges as a versatile tool for quantifying crucial metabolites in clinical oncology, including glucose, lactate, glutamate, glutamine, and characterizing IDH mutations. Its potential applications in this domain are broad, spanning diagnostic profiling, treatment response monitoring, and the identification of novel therapeutic targets across diverse cancer subtypes.

## Introduction

Radioisotopes are widely used as tracers not only to study chemical processes but also to evaluate metabolism *in vivo* when used alongside methods for molecular imaging like PET and SPECT/scintigraphy. The substantial interest in radiotracers in molecular imaging is evident; there has been much research in the field to develop, register, and use radiotracers. Indeed, molecular imaging with a radiotracer offers many advantages, including a high target to non-target ratio for evaluating metabolic pathways of interest. To date, ^18^F-fluorodeoxyglucose (FDG) is one of the most used radiotracers in PET imaging. Nevertheless, while FDG-PET is useful for assessing glucose uptake, it has limitations^1–4^ including that it involves ionizing radiation and does not provide information on downstream metabolism.

Among the different imaging modalities, MRI is a potent noninvasive modality that provides not only a great amount of anatomical information, with different anatomies distinguished by their differing contrast enhancement on acquired images, but also important quantitative information, without the use of ionizing radiation. For example, MRI perfusion imaging provides hemodynamic information, MRI tractography provides structural information, and diffusion-weighted MRI provides functional information. Building on the concept of MRI, which relies on the high natural abundance of water protons within biological tissue, a related technique, proton (^1^H) MR spectroscopy (MRS), was developed to evaluate metabolism in diseased and healthy conditions through the analysis of high natural abundance hydrogen protons (^1^H), showing itself to be a powerful noninvasive and nonionizing method for this purpose. Subsequently, to address the limitation of ^1^H MRS to study characterize highly specific metabolic parameters, multinuclear MRS was introduced, utilizing labelled nonradioactive substrates such as ^13^C, ^2^H, and ^15^N^5–8^ to provide more specific information on the structure and conformations of biomolecules such as proteins and nucleic acids that have a low natural abundance within biological tissue but that can be increased through enrichment with spin labels.

Cancer cells develop alternative metabolic pathways to generate energy, a phenomenon that has been known since the proposal of the Warburg hypothesis in 1927.^9^ Therefore, in the field of oncology, the assessment of metabolism is crucial for diagnosis, prognosis, and treatment evaluation, given that most cancer cells exhibit dysregulated basal metabolism.^10^ To date, nuclear MRS studies have been conducted typically in the form of *in vitro* investigations of proteins and nucleic acids. Hyperpolarized MRS is an *in vivo* technique that applies dynamic nuclear polarization (DNP) techniques to increase nuclear polarization and thereby enhance the sensitivity for detecting metabolites such as ^13^C.^11–13^ Hyperpolarized MRS has shown great potential in preclinical and clinical applications for studying cancer metabolism with high specificity compared to ^1^H MRS. However, for metabolites with a short lifetime of polarization, hyperpolarized MRS has to be completed within a few seconds of dissolution.^3^^,^^14^ Additionally, DNP technique is complex and costly which has restricted its use in wider clinical settings.^15^

In this context, deuterium (^2^H) metabolic imaging (DMI) can offer an alternative approach to studying metabolic processes in vivo while overcoming the limitations of hyperpolarized MRS. DMI is an emerging noninvasive imaging option that combines traditional MRS with deuterium-labelled (^2^H) substrates administered orally or intravenously. DMI can generate three-dimensional or two-dimensional metabolic maps that provide insight into spatial tissue biochemistry, in addition to allowing for co-registration with anatomical images based on conventional anatomical MRI.^16^^,^^17^ Prior to enrichment with labelling, ^2^H has only 0.01% natural abundance compared to 100% of ^1^H. However, after enrichment, ^2^H spectra include signals from the deuterium-enriched substrate and its metabolic end products can be detected with minimum background signal.^17^ Notably, compared with hyperpolarized nuclei, ^2^H has a higher natural abundance within biological tissue and longer relaxation times. DMI may therefore be used to study a wider range of metabolites and metabolic pathways. In addition, while hyperpolarization requires a hyperpolarizer, DMI can simply be performed using standard clinical MRI equipment with MRS capability. Also, deuterated compounds do not incur safety concerns as ^2^H is a stable isotope of hydrogen, along with ^1^H. However, DMI offers lower sensitivity compared to hyperpolarized MRS,^3^^,^^14^^,^^18^ which may affect its ability to detect metabolites at lower concentrations.

Other specific advantages of DMI include (1) The possibility of studying metabolic substrates and metabolites using a nonradioactive labelling method, unlike molecular imaging exams that rely on radiotracers. (2) The ability to study different substrates and metabolites simultaneously, whereas radiotracer-based imaging methods may show energy photopeak overlap in labelling methods (which commonly occurs in conventional emission-based nuclear medicine) or have the same energy (511 keV, in the case of PET studies).^19^

**Figure 1.**
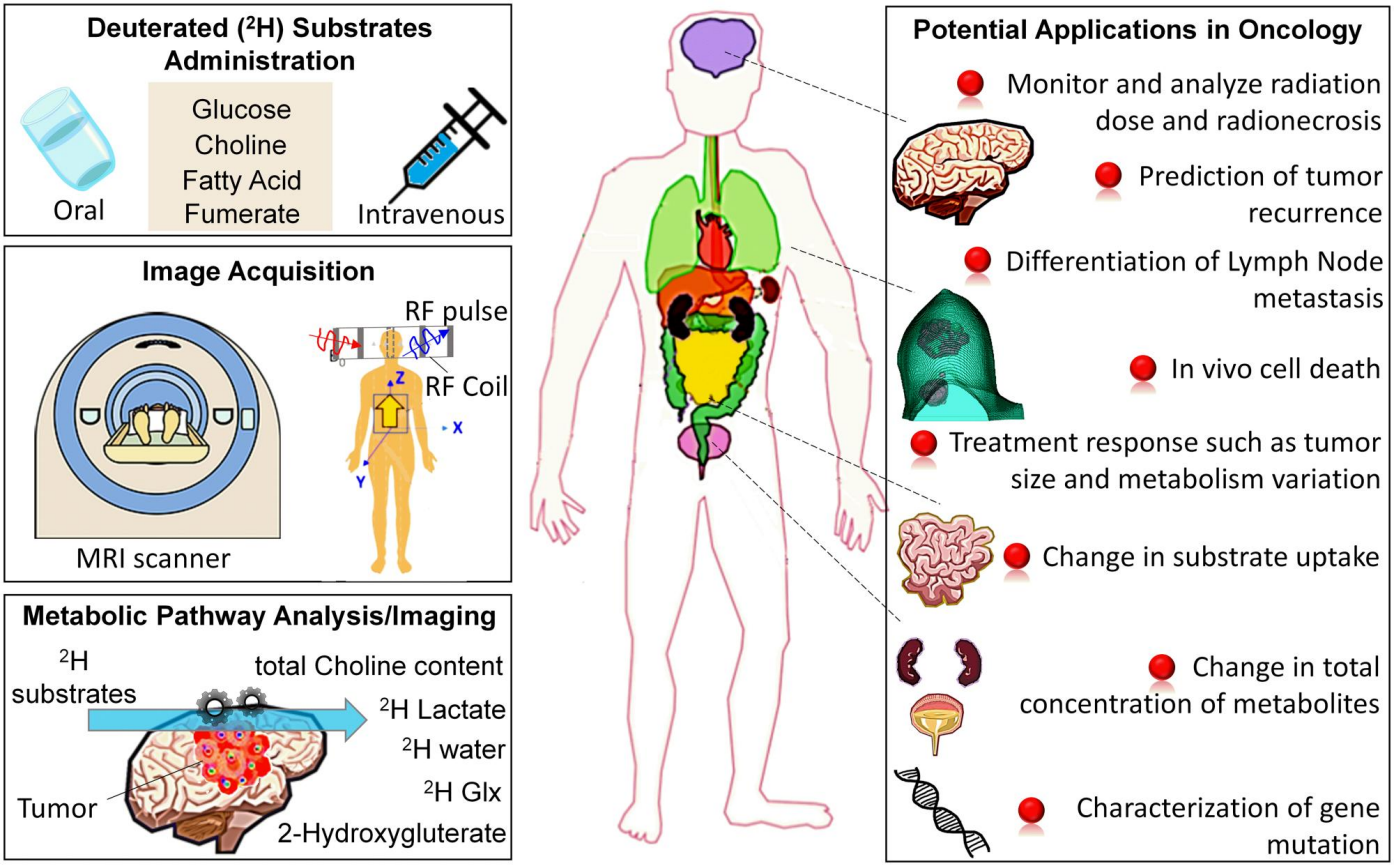
An overview of deuterium metabolic imaging protocol and its potential applications in preclinical and clinical oncology research. Abbreviations: Glx = glutamate and glutamine peaks; RF = radiofrequency.

While DMI has been explored across various diseases including cancer, neurodegenerative disease, stroke, and diabetes, the focus of this review will be on the possible applications of DMI in the oncologic field in particular, which has garnered increasing interest in recent years. In this review, we will cover 3 main areas: (1) technical considerations, including the strengths and limitations of DMI; (2) the main substrates and metabolites that can be analysed with DMI; and (3) the potential applications of DMI in oncology. Figure 1 provides an overview of the DMI protocol and its potential applications in oncology.

## Technical considerations

DMI involves the administration of deuterium-labelled substrates, either orally or intravenously, followed by MRS acquisition of a selected region of interest (ROI). MRS acquires chemical spectra from a selected tissue ROI that can prove useful in the clinical setting. The chemical spectra acquired are reflective of the individual metabolites within ROI, with different metabolites resonating at different positions within the spectra and exhibiting different resonant peaks based on their underlying chemistry. The area under a given metabolite peak is proportional to the concentration of the metabolite. MRS studies have been performed at high magnetic fields (3-7 T) for clinical applications^20^ and even higher magnetic fields (11.7-14.1 T) in preclinical applications.^21^ The specific technical considerations for DMI are detailed below.

### Deuterium labelling

Deuterium-labelled substrates can be administered in a variety of forms, including deuterated water, deuterated glucose, deuterated choline, and deuterated fatty acids.^2^ The selection of the substrate relies on the investigated metabolic pathway. Additionally, studies have used deuterated fumarate substrate to monitor *in vivo* cell death after cancer treatment.^22^ Some initial study protocols have been conducted using ^2^H-labelled glucose as the oral agent to image the ROI, with MRS performed in the morning after overnight fasting and immediately after oral tracer administration using deuterium-labelled glucose ([6,6′]-^2^H-Glc; 0.8 g/kg body weight in 200 mL water).^23^

### MRS acquisition

MRS is carried out using a standard MRI scanner with a specialized radiofrequency (RF) coil tuned to the frequency of deuterium. MRS acquisition is typically done using a point-resolved spectroscopy sequence or a stimulated echo acquisition mode sequence. Shimming may not be crucial for deuterium MRS as there is no water signal to worry about. Of note, in contrast to ^1^H MRS, deuterium MRS can be used to study downstream metabolites such as lactate, glutamate, glutamine, and indirectly IDH gene mutations from increased glycolysis in tumours (see the section “Main Substrates and Detectable Metabolites”), simply through the administration of oral deuterated lactose or glucose. Since deuterated compounds are not radiolabelled compounds, they are safe and ideal for longitudinal treatment monitoring. Clinical deuterium MRS studies in humans have been typically carried out at a magnetic strength of 3-7 T.

### RF coils

To perform deuterium MRI, specialized RF coils are required. These RF coils are designed to transmit RF pulses and receive the signals from deuterium nuclei in the subject being imaged. Deuterium has a different resonance frequency compared to protons, so the RF coils need to be tuned to the appropriate frequency to interact with deuterium nuclei. The coil’s design and tuning will depend on the desired imaging or spectroscopy parameters. The size and geometry of the RF coil are critical for efficient RF energy transmission and reception and this can be the same as vendor-based proton coils. DMI also requires dedicated ^2^H RF coils for MR machines. For humans, RF coils of sufficient quality for DMI at 7 T to diagnose tumours in the brain have been presented that appear to offer a spatial resolution comparable to that of FDG-PET.^16^ Recently, dedicated human ^2^H body coils were also shown to be able to record DMIs, such as those of the liver after [6,6′-^2^H_2_] glucose application.^16^

### Data analysis

Similar to the analysis of spectra acquired by ^1^H MRS, spectra acquired by deuterium MRS at each voxel of the ROI are analysed using specialized software that identifies spectral peaks corresponding to the deuterium-labelled substrates and their metabolites. In *in vivo* studies, when there is no overlap of metabolite peaks within the spectrum, quantification can be achieved by calculating the area under the peak using either the internal water reference method or the external phantom reference method.^24^^,^^25^ At clinical field strengths, such as 3 T, ^1^H MRS of the brain, especially in the presence of various neurological diseases and tumours^26^ typically exhibits overlapping or closely spaced peaks. In these situations, accurate quantification is often performed using the linear combination model (LC model),^27^ which fits the spectrum to a basic set of known metabolite spectra generated under the same experimental conditions. While there are several other methods available for fitting overlapping spectra, but LC model is the most widely used in clinical research.^24^^,^^25^ The quantification of spectra can provide additional information on metabolic rates, metabolic fluxes, and metabolic pathways. MRS metabolite maps from all the voxels within the ROI can be overlaid with anatomical MRI to allow spatial visualization (ie, via metabolic maps). Metabolic maps can provide insights into metabolic pathways and their regulation in healthy and diseased states.

### Repeatability and reproducibility

In the clinical setting, accurate and consistent quantitative measurements are essential for achieving reliable diagnosis, assessing disease progression, and evaluating treatment response. Quantitative biomarkers need to be measured multiple times to assess the experimental variation error limits. A study by Niess et al at 3 T and 7 T^23^^,^^28^ highlighted the importance of multicentre clinical studies assessing repeatability and reproducibility to ensure the accuracy, reliability, and consistency of quantitative measurements, leading to better patient care across the institutions. In their study, an interesting comparison between direct and indirect deuterium-labelled metabolic imaging using specialized 7 T ^2^H DMI protocol and standard 3 T ^1^H quantitative exchange label turnover (QELT) MRSI protocol was also performed for the healthy brain in the same patients.

## Main substrates and detectable metabolites

### Choline

**Figure 2.**
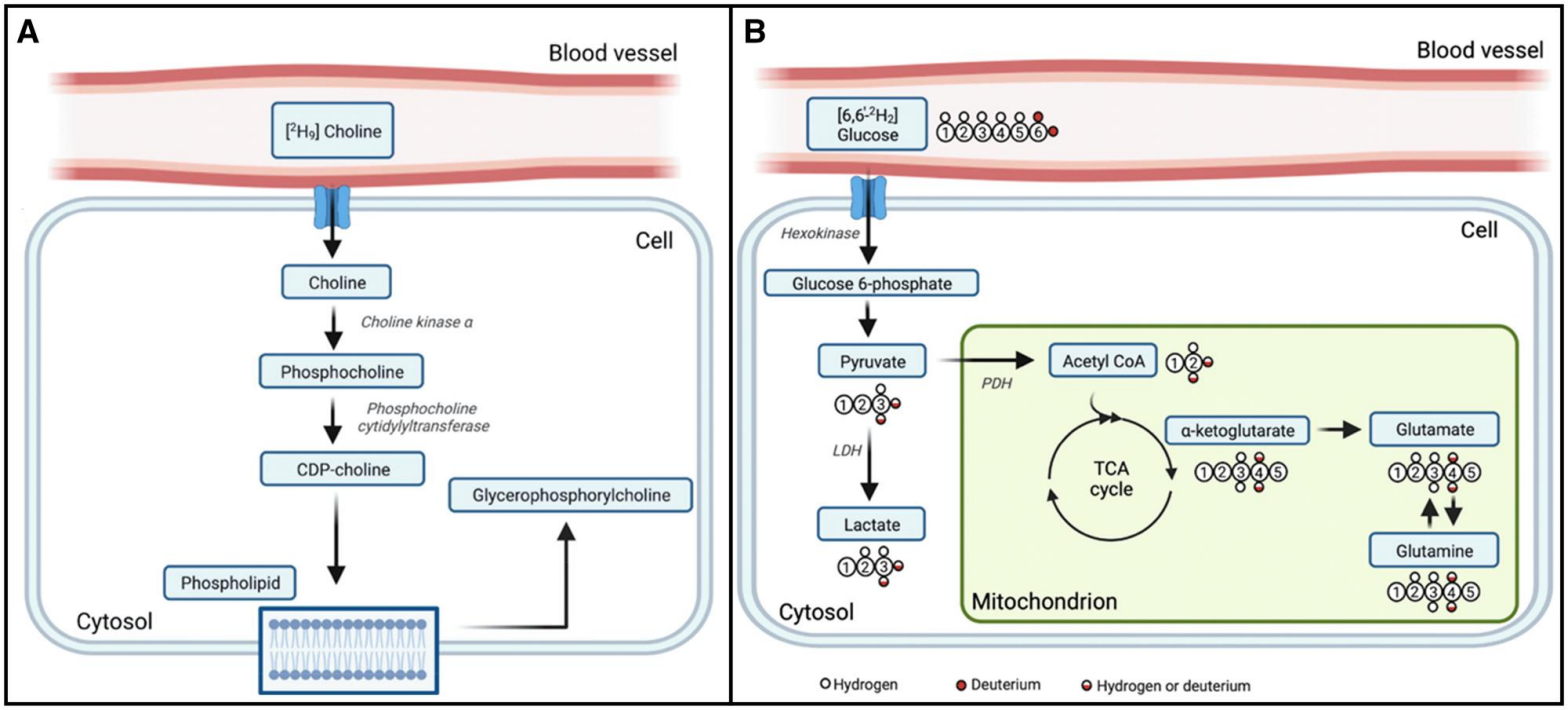
Metabolic products of (A) [^2^H_9_] choline: choline transporter-like protein 1 is responsible for bringing choline into cells. Cholinekinase α then phosphorylates choline to create phosphocholine. Next, phosphocholine cytidylyltransferase then transforms phosphocholine into CDP-choline, which is utilized in the synthesis of phospholipids. Phospholipid degradation results to glycerophosphorylcholine production, and (B) [6,6′-^2^H_2_] glucose: First, cell absorbs glucose with the aid of glucose transporters, where it undergoes phosphorylation to create glucose 6-phosphate (G6P). Hexokinase, the first enzyme in the glycolytic pathway, catalyses this event. Following a sequence of metabolic processes, G6P is converted into pyruvate, which is then either reduced to produce lactate in the cytosol, where it is catalysed by lactate dehydrogenase, or transformed into acetyl-CoA by the mitochondrial pyruvate dehydrogenase complex in the mitochondria. After then, acetyl-CoA enters the TCA cycle and goes through a sequence of oxidation reactions that eventually produce ATP and TCA cycle intermediates, which are the beginning points of several biosynthetic pathways. One of these intermediaries, α-ketoglutarate, is quickly exchanged with glutamate in a reaction that is catalysed by glutamate-pyruvate transaminase. In an irreversible reaction mediated by glutamine synthetase, glutamate can be transformed to glutamine, which can then be broken down into glutamate in a second irreversible reaction mediated by glutaminase. Abbreviations: acetyl-CoA = acetyl coenzyme A; ATP = adenosine triphosphate; CDP = cytidine diphosphate; TCA = tricarboxylic acid. Adapted and reprinted under a Creative Commons License from Low et al^2^

The development of malignant neoplasms is a complex process that entails the ability of cells to adapt and thereby recruit and regulate host stromal cells in order to establish a tumour microenvironment that sustains the survival of neoplastic cells. This recruitment strategy reduces the response of the malignant neoplasm to conventional treatment. Cancer cells are characterized by their genomic and phenotypic plasticity, which provides them with a multitude of redundant pathways, as well as genomic and proteomic diversity.^10^ One hallmark of cancer development is the activation of choline metabolism, also known as the “cholinic phenotype.” This phenomenon has been detected using MRS^29^^,^^30^ and confirmed in different clinical studies.^24^^,^^31^ In fact, higher levels of phosphocholine (PCho) and total choline-containing substances (tCho) have been linked to abnormal choline metabolism across several types of cancers. This fact has significant clinical ramifications, the most evident of which is the importance of noninvasive detection of aberrant choline metabolism through MRS, both at diagnosis and staging. In addition, there is the possibility of exploring choline metabolism for the evaluation of treatment response and for the identification of new targets for treatment.^32^Figure 2A illustrates an overview of biochemical products produced by cells from extracellular [^2^H_9_] choline.^2^ The choline substrate from blood vessels is taken up by cells with the aid of choline transporter-like protein 1. Within the cell, the PCho is catalysed by enzyme choline kinase α (ChoKα). Furthermore, PCho is also catalysed to cytidine diphosphate-choline (CDP-choline) by the enzyme phosphocholine cytidylyltransferase, which is used for phospholipid synthesis. Further, the breakdown of phospholipids leads to glycerophosphorylcholine. The other possible pathways for choline metabolism can be found in Katz-Brull et al^33^

DMI may be advantageous over currently used noninvasive techniques for the assessment of choline metabolism, as it has the potential to provide more specific results in addition to being easily implemented within current clinical multiparametric MRI protocols. Regarding the use of DMI to assess the cholinic phenotype, Veltien et al^19^ showed that DMI had the ability to detect the uptake and conversion of choline, when assessed alone as well as when assessed simultaneously with glucose, after the intravenous administration of [^2^H_9_] choline alone or together with [6,6′-^2^H_2_] glucose, respectively, in mice implanted with human renal carcinoma cells. They observed the broadening of choline signals over time as a result of the production of labelled choline metabolites. The spectrum also revealed an increase in deuterated water signals and labelled lactate signals in the tumour and in case of intravenous administration of both choline and glucose substrate. However, the lactate signals were absent in the tumour in case of infusion of only choline substrate. In another animal study,^34^*in vivo* DMI data collected with infusion of ^2^H-choline, in RG2-bearing rats exhibited high total choline signal in brain tumours but not in normal tissue.^2^^,^^34^ The study revealed high tumour-to-brain contrast maps of labelled choline 24 h after substrate infusion. In yet, another animal study ^2^H-labelling strategies of choline and high-resolution ^2^H NMR in tumour tissue extracts revealed delayed choline-containing metabolites 24 h after intravenous infusion of deuterated choline substrate.^35^

### Lactate

During the initial stages of tumour growth, regions with low oxygen partial pressures emerge; subsequently, in order to ensure a continuous supply of energy supply, glycolysis is induced. Indeed, Otto Warburg first observed in the mid-1920s that tumour processes led to abnormal glucose metabolism, whereby tumour cells preferentially convert pyruvate to lactate, even in the presence of oxygen, a phenomenon now known as the “Warburg effect” or aerobic glycolysis.^9^ The increased concentration of lactate resulting from the Warburg effect has been associated with more aggressive tumour biology.^36^^,^^37^ This is because increased lactate production leads to acidification of the extracellular environment, which in turn promotes the invasion of adjacent structures and metastasis, while inhibiting antitumour immune response^38^ and enabling resistance to anti-neoplastic agents,^38–40^ chemotherapy,^38^^,^^41^ and radiotherapy.^38^^,^^42^

There are clinically well-established methods for characterizing glycolytic metabolism, including PET. However, ^18^F-FDG, the main PET radiotracer, is not able to allow proper compartmentalization of the glycolysis metabolic pathway or assessment of downstream glycolytic metabolism products such as lactate.^43^ Therefore, for the detection and characterization of lactate specifically, ^1^H MRS has been used for this purpose using single-shot selective MQC (selMQC) editing methods^44–47^ or J-difference editing (MEGA) methods.^48^ These sequences were used to detect lactate by suppressing the co-resonating lipids. Though selMQC is a single-shot method that can detect lactate by suppressing lipids, this method has the disadvantage of detecting only 50% of the signal by choosing a coherence transfer pathway of zero-quantum to double quantum. While single-shot DQF methods offer advantages in motion mitigation compared to difference editing lactate sequences, the use of lactate sequences presents a promising biomarker for evaluating tumour characterization. However, single-shot DQF techniques only detect half of the lactate signal, posing a sensitivity challenge in MRS scans of smaller lesion volumes.

**Figure 3.**
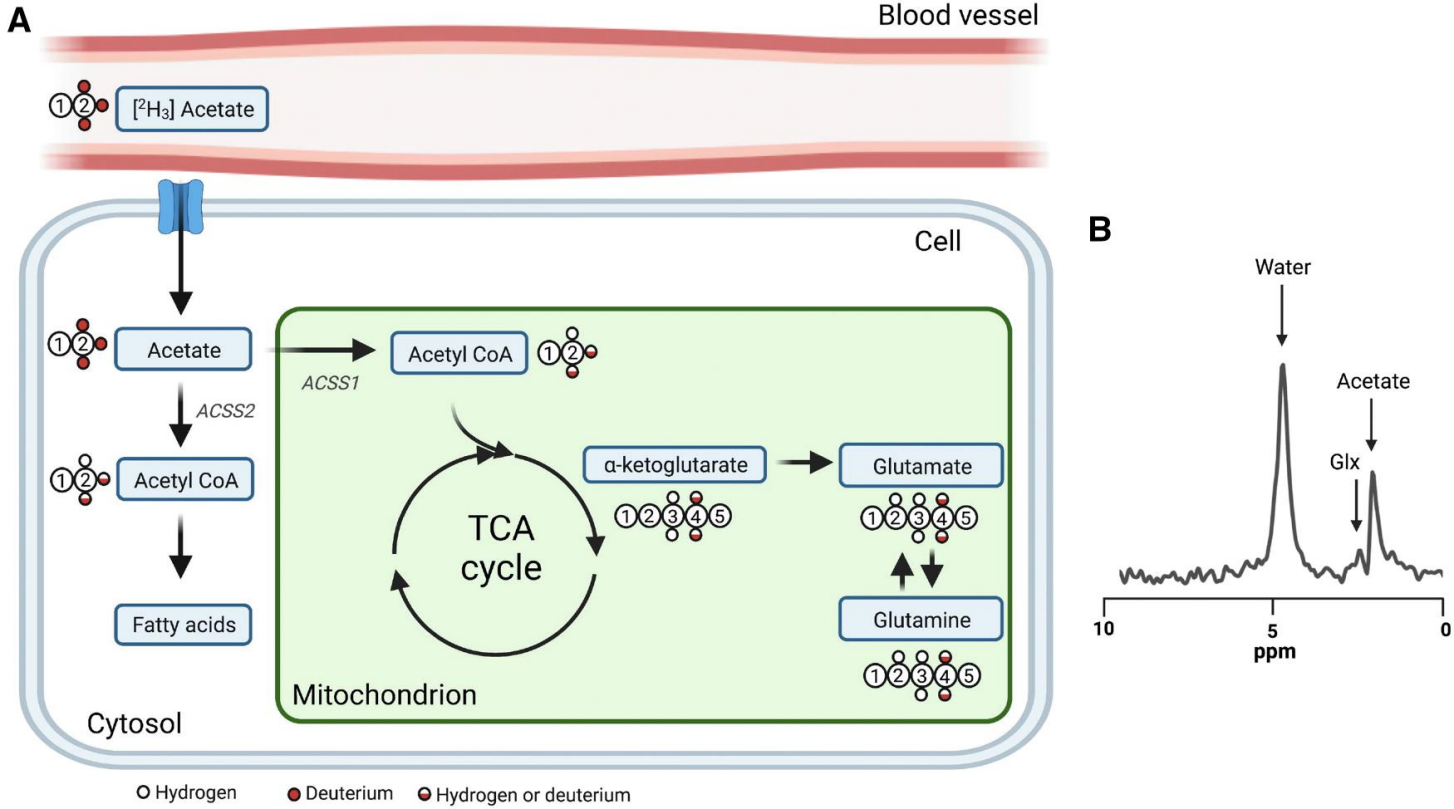
Metabolic products of (A) [^2^H_3_] acetate: monocarboxylate transporters bring acetate into the cell, where it is converted to acetyl-CoA by acetyl-CoA synthetase 1 (ACSS1) in the mitochondria and by ACSS2 in the cytoplasm. While acetyl-CoA in the cytosol can be utilized in the production of fatty acids, it enters the TCA cycle in the mitochondria. (B) Representative spectrum acquired from the brain after intravenous injection of 2 g/kg [^2^H_3_] acetate in a rat bearing an orthotopically implanted patient-derived glioblastoma xenograft. Glutamate and glutamine (Glx) are labelled via α-ketoglutarate in the TCA cycle. Abbreviations: acetyl-CoA = acetyl coenzyme A; TCA = tricarboxylic acid. Reprinted under a Creative Commons License from Low et al^2^

Regarding the use of DMI to characterize lactate *in vivo*, recently De Feyter et al^16^ investigated DMI in a rat glioma model as well as in 2 patients with glioblastoma multiforme who received [6,6′-^2^H_2_] glucose orally. DMI identified areas within the tumour lesion with greater lactate concentrations than healthy brain regions both in the rats and 2 patients. Moreover, the lactate-to-glutamine ratio enabled spatial visualization of the Warburg effect. It is worth noting that evaluating the lactate-to-glutamine ratio with DMI may be practical over evaluating pyruvate with hyperpolarized ^13^C MRI,^18^ since deuterated compounds used in DMI are somewhat less expensive than hyperpolarized ^13^C-labelled compounds and long-lived signals, metabolically equivalent to unlabelled substrates, and not radioactive.^49^ To investigate the glucose-lactate metabolism pathway in tumours, DMI typically uses deuterated oral [6,6′-^2^H_2_] glucose as substrate, which allows for the detection of its downstream metabolites, predominantly [3,3′-^2^H_2_] lactate, but also [^2^H_3_] lactate.^18^ In fact, the oral administration of DMI substrate is believed to be more convenient protocol for patients. Figure 2B illustrates an overview of biochemical products produced by cells from extravasation of extracellular [6,6′ ^2^H_2_] glucose^2^ from blood vessels. The [6,6′-^2^H_2_] glucose is one of the widely used substrates that leads to various intermediate labelled metabolites in the glycolytic pathway such as pyruvate, lactate, α-ketoglutarate, glutamate, and glutamine. The loss of ^2^H isotope in the glycolytic pathway and labelling patterns are detailed in Kaggie et al^50^ On the other hand, they also^16^ investigated DMI in a rat glioma model that was infused with [^2^H_3_] acetate substrate. After infusion of [^2^H_3_] acetate substrate, DMI of *in vivo* rat glioma exhibited higher level of labelled acetate and lower level of labelled glutamate and glutamine (Glx) spectra for tumour. Whereas, in the normal-appearing brain tissue, a homogeneous distribution of acetate oxidation was observed. Figure 3A illustrates an overview of biochemical products produced by cells from extracellular [^2^H_3_] acetate,^2^ and Figure 3B depicts a representative deuterium spectra of rat glioma model exhibiting a higher level of label acetate compared to Glx peak.

### Glutamine and glutamate

One of the main metabolic abnormalities present in the majority of cancer cells is aberrant glutamine metabolism, which is second only to abnormal glucose metabolism. Glutamine is the most prevalent amino acid in plasma and plays a crucial role in essential metabolic pathways for cell survival and proliferation, including adenosine 5′-triphosphate (ATP) generation, protein synthesis, nucleic acid synthesis, and lipid synthesis. The catabolism of glutamine occurs via the mitochondrial enzyme glutaminase, which hydrolyses glutamine into glutamate and ammonia. Glutamate deamination yields α-ketoglutarate, which is one of the tricarboxylic acids (TCA) cycle’s intermediates. Cancers such as neuroblastoma, lymphoma, renal carcinoma, and pancreatic adenocarcinoma rely on abnormal glutamine metabolism to support their growth and survival.^51^ Thus, glutamine addiction is emerging as an important hallmark of many cancers.^52^ Considering that inhibiting glutamine metabolism can limit tumour growth and promote antitumour immune activity, the glutamine pathway may have a potential role in assessing progression and response to treatment.^53^^,^^54^

Regarding the use of DMI to evaluate glutamate and glutamine *in vivo*, the study De Feyter et al^16^ mentioned above demonstrated that DMI was able to identify the peaks of glutamate and glutamine in a rat model and in humans with glioblastoma multiforme as well as allow the evaluation the lactate-to-glutamine ratio. They also pointed out that infusion of [6,6′-^2^H_2_] glucose substrate led to a reduction of Glx labelling and an increase in lactate labelling in tumour for both patients and in the tumour model. This observation indicates a reduction in TCA activity and an increase in glycolytic activity in tumour. The production of labelled glutamate and glutamine intermediates in glycolytic pathway is detailed in Figure 2B.^2^

### ^2^H labelled substrates (pyruvate, fumarate, fructose, and heavy water)

Different deuterated substrates of pyruvate, fumarate, fructose, methylglucose, and heavy water were employed in cancer investigations, including monitoring the growth of tumours, the response to therapies, the quantification of cell death and radionecrosis, and the uptake of metabolizable and non-metabolizable substrates by cells (see Table 1). The substrate [U-^2^H]-pyruvate has been identified in preclinical tumour models^56^ as an early reporter for treatment response with a notable reduction in lactate production prior to observable changes in tumour volume. This reduction in lactate production was linked to lower expression of telomerase reverse transcriptase expression (TERT) in the tumour due to TERT inhibition drugs, as TERT expression is a potential marker for cell proliferation. The study also revealed the production of lactate in tumours when infused with [U-^2^H]-pyruvate, but no lactate signals were observed before the infusion of pyruvate and in normal-looking tissue. In another study,^65^ intravenous injection of [3,3′,3″-^2^H_3_]-pyruvate substrate revealed a relatively faster conversion rate of pyruvate to lactate in a tumour model of pancreatic cancer as compared to intravenous injection [6,6′-^2^H_2_]-glucose. Overall, the study indicated that deuterated lactate signals from tumours are long-lived if converted from glucose substrate (range 45-60 min) then that was converted from pyruvate substrate (∼6 min). The specificity of lactate signals from tumours is weak in case of pyruvate substrate as compared to glucose substrate, but a high contrast for tumour can be achieved by taking lactate/pyruvate ratio. In related studies on pancreatic tumour models by the same group,^63^ glucose substrate-based improved DMI imaging technique was proposed to enhance the signal-to-noise ratio of metabolic signals as compared to conventional chemical shift imaging method. The group^64^ also showed glucose substrate-based DMI could be a useful technique to differentiate between pancreatitis and pancreatic cancer (see Table 1). In yet another study on the pancreatic tumour model^61^ revealed that deuterated lactate signals are predominantly localized in the tumour. However, deuterated water signals are pronounced throughout the abdomen and tumour.

**Table 1.** Summary of selected clinical and preclinical cancer studies using deuterium metabolic imaging.

Methods	Study	Clinical/preclinical	Patients/xenografts	Application	MRI vendor (field strength)	Remarks
^2^H-labelled glucose substrate DMI	De Feyter et al, 2018^16^	Clinical	Brain (Glioblastoma multiforme)	Diagnosis and prognosis	Bruker (4.0 T)	In a study of 2 patients, 60-75 min after oral intake of substrate, DMI exhibited homogeneous distribution of ^2^H-glucose over slices but lesser levels of ^2^H-labelled Glx and a higher concentration of ^2^H-labelled lactate in tumour than normal tissue.
^2^H-labelled glucose substrate DMI	Niess et al, 2023^28^	Clinical	Brain (healthy)	Reproducibility	Siemens (7.0 T and 3.0 T)	In a cohort of healthy subjects, 1 h after oral intake of substrate, it was shown that indirect clinical 3 T ^1^H quantitative exchange label turnover MRS protocol could reproduce the absolute concentrations of downstream glucose metabolites and dynamics of glucose uptake compared to direct deuterium-labelled metabolic imaging using specialized 7 T ^2^H DMI protocol.
^2^H-labelled glucose substrate DMI	De Feyter, 2018^16^	Preclinical	Glioma (rat)	Diagnosis and treatment response	Bruker (11.7 T)	DMI exhibited marked decrease in Glx peak and high level of lactate in tumour compared to normal brain. They also evaluated the treatment response of tumour using DMI post administration of dichloroacetate. Treatment appears to reflect reduction in tumour lactate/Glx parametric ratio maps.
^2^H-labelled acetate substrate DMI	De Feyter, 2018^16^	Preclinical	Glioma (rat)	Diagnosis	Bruker (11.7 T)	DMI exhibited higher level of labelled acetate and lower level of labelled Glx in tumour.
^2^H-labelled glucose substrate and ^1^H qMRS	Rich et al, 2020^55^	Preclinical	Glioma (rat)	Diagnosis	Bruker (9.4 T)	The change in tumour lactate concentration (ie, pre-post administration of the substrate) was quantified using ^1^H MRS. After 60 min of substrate infusion, a marked reduction in the lactate peak was observed suggesting considerable ^2^H labelling of lactate. This change was not significant in normal tissue.
^2^H-labelled pyruvate substrate DMI	Batsios et al, 2022^56^	Preclinical	Glioblastoma, glioma, hepatocarcinoma (mice)	Tumour proliferation and therapy response	Varian (14.1 T) and Bruker (3.0 T)	In this study, the TERT expression in tumour was monitored which is identified as tumour proliferation factor. Here, the lactate production from substrate in case of TERT+ tumour is significantly higher than TERT healthy tissue. Whereas, the use of a TERT inhibitor as a therapy surrogate revealed significant reduction in lactate production at early time-points prior to observable changes in tumour volume. Thus, revealed pyruvate substrate could be an early predictor for treatment response.
^2^H-labelled glucose substrate DMI	Ge et al, 2022^57^	Preclinical	Glioblastoma (mice)	Radionecrosis	Agilent (11.74 T)	Tumours showed an intense and statistically significant Warburg effect compared to oxidative glucose metabolism in radionecrosis.
^2^H-labelled choline substrate DMI	Ip et al, 2023^34^	Preclinical	Glioma (rat)	Diagnosis	Bruker (11.74 T)	DMI exhibited high total choline signal in tumours compared to normal tissue.
^2^H-labelled -3-O-Methylglucose (OMG) substrate DMI	Hartmann et al, 2021^58^	Preclinical	Breast (rat)	Uptake of nonmetabolizable substrate	Bruker (7.0 T)	Time evolution of deuterium signals was measured to determine the washout of OMG. OMG is a nonmetabolizable glucose analogue, considered nontoxic for diagnostic purposes, and a promising candidate for assessing washout kinetics and glucose uptake.
^2^H-labelled Fumarate substrate DMI	Hesse et al, 2021^59^	Preclinical	Breast, lymphoma, colorectal (mice)	Treatment response (detecting cell death)	Agilent (7.0 T)	Tumours exhibited increase in ^2^H labelled malate production in mice treated with drug (Epitosoid) in vivo as compared to untreated mice. The production of malate is an indication of cell death of tumour confirmed by immunohistochemistry and NMR techniques.
^2^H-labelled Fructose and glucose substrates DMI	Zhang et al, 2023^60^	Preclinical	Liver (mice)	Diagnosis	Bruker (9.4 T)	There was a change in tumour spectra as a function of time following post infusion of ^2^H-labelled fructose or glucose. Net lactate signal was visible after time integral of spectra between 30 and 60 min.
^2^H-labelled glucose substrate DMI	Markovic et al, 2021^61^	Preclinical	Pancreatic (mice)	diagnosis	Bruker (15.2 T)	DMI maps indicated decay of the injected glucose, and the concurrent generation of deuterated water and lactate peaks. The lactate was found localized only in the tumour, whereas deuterated water imaged throughout the abdomen (ie, kidney and bladder) with peak value in tumour.
^2^H-labelled water substrate DMI	Asano et al, 2023^62^	Preclinical	Pancreatic and colorectal (mice)	treatment response	Medalist (1.5 T)	Tumour models were individually treated with radiation or chemotherapy. DMI images of treated and untreated mice were analysed up to 7-10 days after drinking of ^2^H-labelled water to evaluate the treatment response. In both treatment methods, the deuterated water signals in tumour decreased with time, along with significant reduction in tumour volume noted in MRI of radiotherapy cohort. The H&E-stained tumour tissue sample revealed larger necrotic region in case chemotherapy cohort but not in radiotherapy cohorts.
^2^H-labelled glucose substrate DMI	Montrazi et al, 2023^63^	Preclinical	Pancreatic (mice)	SNR improvement	Bruker (15.2 T)	This study compared the use of ME-*b*SSFP imaging technique to improve the signal-to-noise ratio of DMI metabolic signals as compared to conventional CSI method. Enhanced sensitivity of metabolites were observed.
^2^H-labelled glucose substrate DMI	Montrazi et al, 2023^64^	Preclinical	Pancreatic (mice)	Differentiation between pancreatic cancer and pancreatitis	Bruker (15.2 T)	This comparison study revealed ME-SSFP imaging technique though improves the signal-to-noise ratio by 5 times than conventional CSI method. There is no lactate concentration was observed for pancreatitis as compared to pancreatic cancer. The study revealed that the deuterated lactate may be a biomarker to differentiate between pancreatitis and pancreatic tumour.
^2^H-labelled pyruvate and glucose substrate DMI	Montrazi et al, 2024^65^	Preclinical	Pancreatic (mice)	Diagnosis	Bruker (15.2 T)	In comparison to glucose substrate, pyruvate substrate indicated faster conversion of pyruvate to lactate in pancreatic cancer. The deuterated lactate from tumours have a longer life generated from glucose substrate as opposed to pyruvate substrate.
^2^H-labelled glucose substrate DMI	Kreis et al, 2020^66^	Preclinical	Lymphoma (mice)	Treatment response	Agilent (9.4 T)	Kinetic model-based fitting of spectroscopic signals revealed a decline in glycolytic flux 24 h after chemotherapy. There was also decline in lactate-to-water ratio and lactate-to-glucose ratio of tumour.
^2^H-labelled choline and glucose substrate DMI	Veltien et al, 2021^19^	Preclinical	Renal (mice)	Diagnosis	Bruker (11.7 T)	Tumour uptake of choline and glucose was analysed or imaged simultaneously after combined infusion of ^2^H-labelled choline and glucose substrates. The time evolution revealed broadening of choline signals, increase in deuterated water signals and lactate signals in the tumour. The lactate signals were absent in tumour for the case of infusion of ^2^H-labelled choline substrate only.

Abbreviations: CSI = chemical shift imaging; DMI = deuterium MRI; Glx = glutamate and glutamine peaks; ^2^H MRS = deuterium MRS; ^1^H MRS = proton MRS; ME-bSSFP = multi-echo balanced steady state free precession; NMR = nuclear magnetic resonance; qMRS = quantitative proton MRS; SNR = signal-to-noise ratio; TERT = telomerase reverse transcriptase expression.

**Figure 4.**
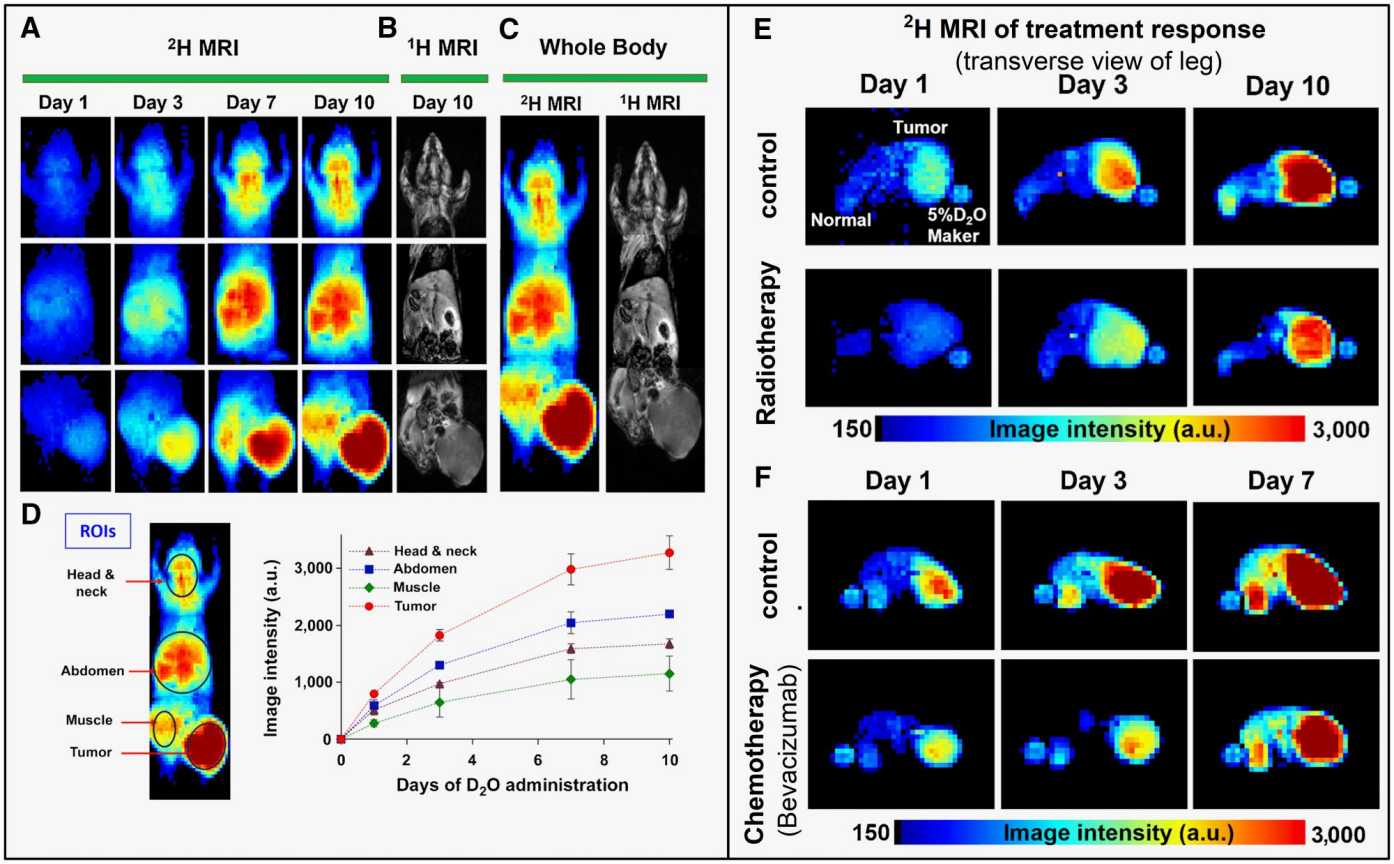
*In vivo* DMI maps of treatment response of tumour bearing mice which has been given unrestricted access to drink D_2_O or heavy water. (A) Coronal deuterium MRI depicting the distribution of ^2^H throughout the body during the administration of D_2_O in mice. After the first exposure to D_2_O, images were taken on days 1, 3, 7, and 10. (B) T_1_-weighted MRI of mouse body parts. (C) The distribution of ^2^H and ^1^H signals gathered throughout the body is displayed in full-body MRI that are obtained by combining the MRI images of 3 body sections. (D) Quantification of ^2^H MRI signals of body tissues. The data show the mean ± SD (*n* = 6). (E) Representative transverse DMI images of the legs of mice bearing MIA PaCa-2 human pancreatic cancer, both during and after D_2_O administration and radiation. Images were collected on days 1, 3, and 10 of irradiation. (F) Representative transverse DMI images of the legs of mice bearing MIA PaCa-2 human pancreatic cancer, both during and after D_2_O administration and bevacizumab treatment. Images were collected on days 1, 3, and 7 of administration. Abbreviations: DMI = deuterium metabolic imaging; D_2_O = heavy water or deuterium oxide. Adapted and reprinted under a Creative Commons License from Asano et al^62^

In yet another study after intravenous injection [2,3-^2^H_2_] fumarate,^59^ the conversion of malate from fumarate or malate-to-fumarate ratio is identified as a key biomarker for quantification of cell necrosis in a tumour model of breast, lymphoma, and colorectal cancer subjected to treatment. Here, fumarate quickly reaches fumarase in necrotic cells through the permeable plasma membrane as compared to the less permeable plasma membrane of viable cell, leading to a significant rise in malate production either within or outside the cell in the necrotic region. Similarly, in another study, DMI maps of mice with xenografted pancreatic or colorectal tumours treated with radiotherapy or chemotherapy were evaluated for up to 7-10 days while unrestricted drinking of heavy water.^62^ Here, the deuterated water signals in the tumour decreased with time in both treatment methods, and the MRI of the radiation group showed a notable decrease in tumour volume. Figure 4 depicts the *in vivo* DMI maps of treated tumour-bearing mice, which have been given unrestricted access to drink D_2_O or heavy water.

The substrate [6,6′-^2^H_2_] fructose on the other hand was used as an alternate DMI probe to overcome the limitation of substrate [6,6′-^2^H_2_] glucose in liver cancer,^60^ since fructose is primarily processed in the liver and deuterated water converted from fructose is likely to be less polluted by whole-body metabolism than glucose. In addition, ^2^H fructose is easily converted to ^2^H Glx in the normal liver, making it possible to characterize the kinetics of ^2^H fructose. This resolves a significant drawback of earlier ^2^H glucose investigations in the liver, which could not reliably identify metabolic flux because of the signals from ^2^H glucose and its overlapped metabolic product (^2^H glycogen). In case of liver cancer, lactate signals were unresolvable in individual time spectra for both substrates. However, the net lactate signal improved after time integral of spectra between 30 and 60 min after injection of substrate. The metabolic pathway of [6,6′-^2^H_2_] fructose is detailed in Markovic et al^60^

## Potential applications in oncology

^1^H MRS is currently used routinely to study altered metabolism in brain tumours, including for the identification of IDH mutations and the assessment of treatment efficacy. Here, we move beyond ^1^H MRS to explore the potential of DMI for various oncologic applications including diagnosis, treatment response monitoring, and discovery of new therapeutic targets. DMI may be used to measure changes in metabolic rates before and after treatment, providing insight into treatment efficacy. DMI may also be used to find metabolic pathways that are different in cancer cells from normal cells, which can be targeted by new therapies. Table 1 presents clinical and preclinical studies with their findings associated with deuterium MRSI measurements/application in oncology.

### Tumour diagnosis and treatment assessment

DMI has been demonstrated as a promising noninvasive imaging modality for tumour diagnosis. By detecting and quantifying metabolic abnormalities associated with tumourigenesis, DMI can aid in the characterization and differentiation of tumours from normal tissue.

#### Central nervous system

**Figure 5.**
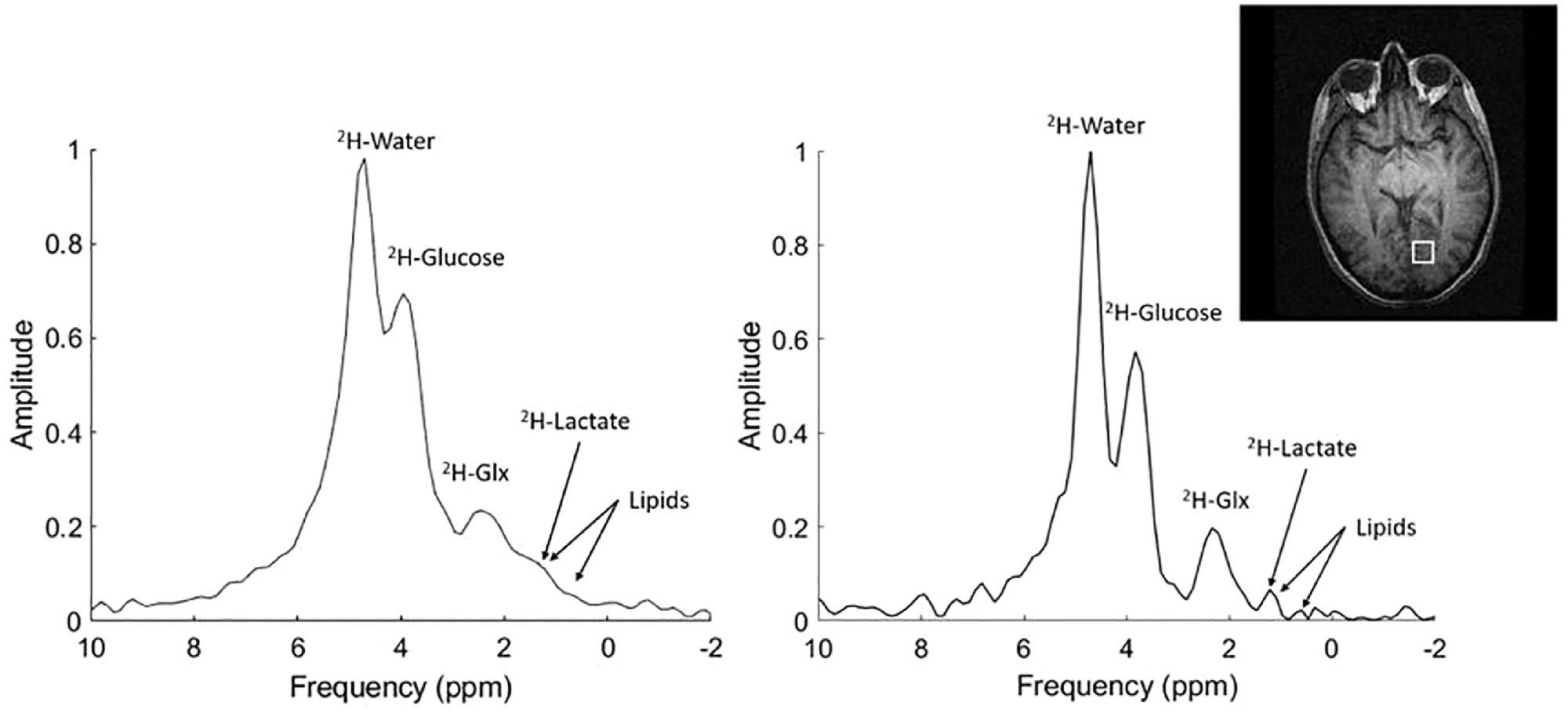
A typical DMI spectra of healthy human brain acquired 90 min after oral administration of ^2^H-glucose using 3 T MRI. Left: whole-brain ^2^H-spectra. Right: Localized ^2^H-spectra acquired from a single midline voxel (3.2 cm isotropic). The localized spectrum demonstrates that ^2^H-water (4.7 ppm), ^2^H-glucose (3.9 ppm), ^2^H-Glx (2.4 ppm), and ^2^H-lactate (1.35 ppm) peaks were more easily detected than on the global spectrum because of line broadening from magnetic nonuniformities. The lipids peak at 0.9 ppm was close to the noise floor. The white box indicates the interpolated region from which the local spectrum was derived. Abbreviations: DMI = deuterium metabolic imaging; Glx = glutamate and glutamine peaks. Reprinted under a Creative Commons License from Kaggie et al^18^

DMI has potential application in a particularly important and challenging scenario: differentiating between tumour radionecrosis and recurrence in the central nervous system. In an investigation performed in a mouse model of radionecrosis, DMI in conjunction with [6,6'-^2^H_2_] glucose infusion provided a substantial dynamic range in distinguishing tumour from radionecrosis and healthy tissue, as tumours showed an intense and statistically significant Warburg effect compared to oxidative glucose metabolism in radionecrosis and control healthy tissue (normal brain).^57^Figure 5 shows the *in vivo* deuterium spectra from a normal whole-brain and from localized volume as indicated.

#### Head and neck

There are only a few studies that have specifically addressed the use of DMI in head and neck tumours, including the study by De Feyter et al^16^ previously mentioned above.

**Figure 6.**
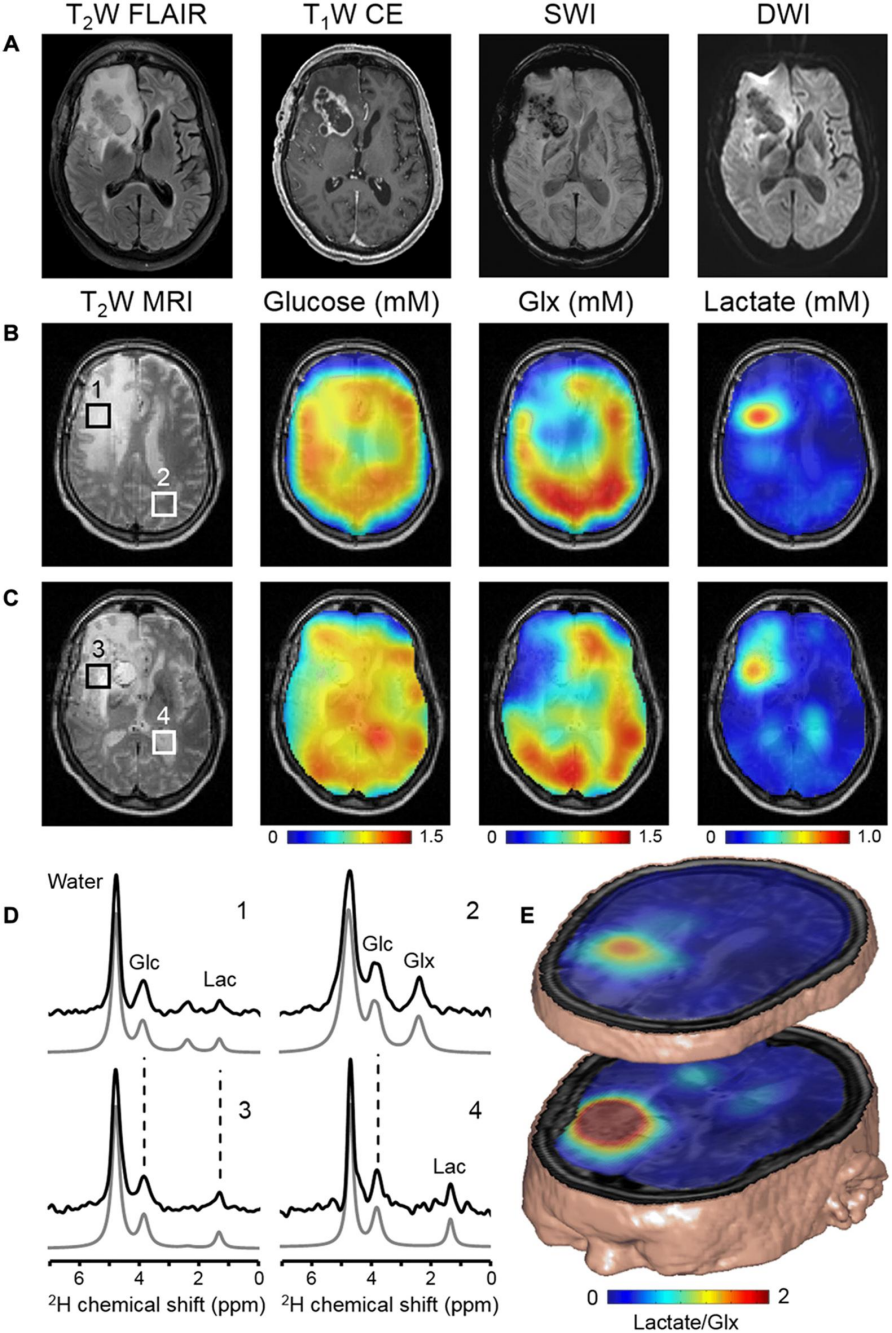
DMI visualizes the Warburg effect in a patient with GBM after oral [6,6′-^2^H_2_] glucose intake. (A) Clinical MRI acquired as standard-of-care in a patient diagnosed with GBM in the right frontal lobe. MRI include (from left to right) T_2_-weighted fluid-attenuated inversion recovery (T_2_W FLAIR), T_1_-weighted contrast-enhanced imaging (T_1_W CE), susceptibility-weighted imaging (SWI), and diffusion-weighted imaging (DWI). The patient (male, 63 years) had undergone subtotal resection of the lesion 9 months before the DMI study. He was undergoing treatment on an experimental protocol involving nivolumab or placebo combined with the standard-of-care chemoradiation with temozolomide, followed by adjuvant temozolomide. (B and C) T_2_W MRI and overlaid DMI maps in 2 slices that contain the tumour lesion. The MRI and DMI data shown in (C) correspond to the slice position of the clinical MRI scans shown in (A). DMI maps show a homogeneous distribution of ^2^H-glucose across the slices but lower levels of ^2^H-labelled Glx and a higher concentration of ^2^H-labelled lactate in the tumour lesion compared to normal-appearing brain. (D) ^2^H NMR spectra from selected locations depicted in the T_2_W MR image, including tissue (1 and 3) within the lesion as seen on T_1_W CE, (2) from normal-appearing occipital lobe, and (4) containing cerebrospinal fluid from the left lateral ventricle. (E) 3D illustration of combined MRI and DMI of the lactate/Glx ratio representing the spatial distribution of the Warburg effect. Abbreviations: DMI = deuterium metabolic imaging; GBM = glioblastoma multiforme brain tumour; Glx = glutamate and glutamine peaks. Reprinted under the terms of the Creative Commons Attribution-NonCommercial license from De Feyter et al^16^

In particular, lactate has been shown by different imaging modalities to be able to yield significant information about head and neck malignancies. For example, exogenous lactate is related to the motility and migration of head and neck carcinoma cell lines in a dose-dependent manner.^67^ Additionally, it has been shown that lactate concentrations are significantly higher in tumours with lymph node metastases when compared to tumours with no metastases.^68^ In the context of treatment assessment with radiotherapy, in head and neck tumours, both *in vitro* and *in vivo* experiments have shown a positive correlation between lactate concentration and the radiation dose required for tumour control in human head and neck squamous cell carcinoma.^42^^,^^69^ On the other hand, in the context of treatment assessment with chemotherapy, DMI of the lymphoma model revealed a decline in glycolytic flux 24 h after chemotherapy.^66^ Additionally, it has been also shown that lactate concentration may be a predictor of recurrence in head and neck squamous cell carcinoma.^70^ Lastly, an indirect method can be employed to monitor the lactate labelling. For instance, ^1^H MRS of glioma tumour model^55^ was investigated post infusion of ^2^H-labelled glucose substrate which has revealed a significant reduction in the lactate peak in ^1^H MRS signals suggesting considerable ^2^H labelling of lactate. Figure 6 shows quantitative maps and a comparison of *in vivo* concentration changes in deuterium spectra between healthy and malignant region of a patient’s brain with glioblastoma multiforme brain tumour.

#### Breast

DMI was shown in Hesse et al’s study^59^ to enable the detection of malate production as well as the evaluation of the malate-to-fumarate ratio in breast tumour. Specifically, the significant increase in malate-to-fumarate ratio in the 5 mice with triple-negative breast cancer xenografts after treatment with a TRAILR2 agonist compared with 3 healthy control mice (*P* < .01). Thus, DMI may prove to be a sensitive and inexpensive method for detecting cell death *in vivo*, offering advantages over current methods for response assessment that are based on variation in tumour size (RECIST—Response Evaluation Criteria in Solid Tumours) or metabolism variation (PERCIST—PET Response Criteria in Solid Tumours).

In another preclinical study Hartmann et al,^58^ DMI of deuterated 3-O-methylglucose (OMG) was demonstrated in a mouse model of breast cancer bone metastases. OMG is a nonmetabolizable glucose analogue, considered nontoxic for diagnostic purposes, and a promising candidate for assessing glucose uptake.

#### Renal tumours

Simultaneous detection of choline and glucose has clinical potential for tumour characterization, especially considering its feasibility in patients. In Veltien et al’s study conducted at an 11.7 T MR field strength,^19^ they examined the uptake and conversion of [^2^H_9_] choline, both alone and in conjunction with [6,6'-^2^H_2_] glucose, through infusion into tumours in mice with human renal carcinoma cells using DMI. They reported tumour concentrations of deuterated choline in the range of 0.3-1.2 mM. Co-infusion of [^2^H_9_] choline and [6,6'-^2^H_2_] glucose showed distinct spectra in tumour MR. As a function of increasing time after infusion, lactate signal was detected, indicating glycolysis.

#### Genitourinary tumours

As mentioned in the preceding section on main DMI substrates and detectable metabolites, Veltien et al showed that DMI was able to detect the uptake and conversion of choline, when assessed alone as well as when assessed simultaneously with glucose, after the intravenous administration of [^2^H_9_] choline alone or together with [6,6′-^2^H_2_] glucose, respectively, in mice implanted with human renal carcinoma cells.^19^

Of note, while not specifically evaluating the use of DMI, several studies have underscored the relevance of metabolites in genitourinary tumours. For instance, MRS is particularly useful for the identification of elevated choline compound levels in prostate cancer. However, studies on the value of MRS in prostate cancer have been mostly based on ^1^H MRI.^71^ It is worth mentioning that the overlap between the choline, creatine, and spermine resonances makes it difficult to individually assess these metabolites with ^1^H MRI. In this context, DMI may emerge as a potential technique to overcome this limitation.

### IDH gene mutation diagnosis

2-hydroxyglutarate (2-HG) is a metabolite that is produced through the abnormal metabolism of glutamine. Mutations in isocitrate dehydrogenase (IDH) genes have been found to lead to an accumulation of 2-HG in some types of cancer, such as glioma, acute myeloid leukaemia, and a few others. Recent studies have also suggested that DMI may have applications in the detection and characterization of certain gene mutations associated with cancer, such as IDH1/2 mutations. These mutations lead to the increased Warburg effect, detected by DMI.^72^ A study with 21 patients underwent DMI at 4.7 T field strength after oral administration of ^2^H-glucose. They have measured the Warburg effect by calculating ratio of deuterated lactate over the sum of glutamate and glutamine.

## Conclusions

In conclusion, DMI is a promising imaging technique within clinical oncology to measure relevant metabolites and provide valuable insights into cancer metabolism. DMI may be used to distinguish malignant from healthy tissue. Additionally, by monitoring variations in metabolic activity over time, DMI may offer insightful data on the effectiveness of therapies and help guide treatment decisions. Yet another possible use of DMI is to identify novel therapeutic targets, such as in tumours with IDH mutations. Despite DMI’s potential, to date, limited research has been done on its application for human cancer diagnosis and prognosis. Our extensive literature review revealed that animal xenografts with various cancer models are the main settings in which DMI has been predominantly explored. Consequently, it is imperative to apply this metabolic imaging technique to human oncologic studies. Further research is needed to optimize DMI protocols and evaluate their clinical utility.
